# High serum ferritin level is an independent risk factor for metabolic syndrome in a Chinese male cohort population

**DOI:** 10.1186/s13098-015-0004-9

**Published:** 2015-02-24

**Authors:** Qin Tang, Zhenfang Liu, Yan Tang, Aihua Tan, Yong Gao, Zheng Lu, Qiuyan Wang, Yingchun Chen, Chunlei Wu, Haiying Zhang, Xiaobo Yang, Zengnan Mo

**Affiliations:** Center for Genomic and Personalized Medicine, Guangxi Medical University, Nanning, Guangxi 530021 China; Guangxi Key Laboratory of Genomic and Personalized Medicine, Nanning, Guangxi 530021 China; Guangxi Collaborative Innovation Center for Genomic and Personalized Medicine, Nanning, Guangxi 530021 China; Hematology Department, The First Affiliated Hospital of Guangxi Medical University, Nanning, Guangxi China; Department of Chemotherapy, The Affiliated Tumor Hospital of Guangxi Medical University, Nanning, Guangxi 530021 China; Institute of Urology and Nephrology, First Affiliated Hospital of Guangxi Medical University, Nanning, Guangxi 530021 China; Department of Occupational Health and Environmental Health, School of Public Health, Guangxi Medical University, Nanning, Guangxi 530021 China

**Keywords:** Metabolic syndrome, Serum ferritin, Central obesity, Hypertension, Triglycerides, High-density lipoprotein cholesterol

## Abstract

**Background:**

Elevated serum ferritin levels have been reported to contribute to metabolic syndrome (MetS). We examined the association of serum ferritin levels with the development of MetS in a representative sample of Chinese male adult population.

**Method:**

The data came from the 2009–2013 Fangchenggang Area Males Health and Examination Survey (FAMHES). We combined a cross-sectional study of 2417 males and a longitudinal study of 857 males who participated in the FAMHES.

**Result:**

The serum ferritin level of MetS was higher than that of nonMetS (median and percentiles 25–75: 447.4 (294.1-612.4) vs. 302.4 (215.0-435.8) ng/ml, *p* < 0.01). A positive correlation between ferritin concentrations and blood pressure (Systolic BP: R = 0.110, Diastolic BP: R = 0.158), waist circumference (R = 0.333), fasting glucose (R = 0.089), triglyceride (R = 0.315) and low high-density lipoprotein cholesterol (R = 0.130) was significant (all *p* < 0.001). Compared with the level of ferritin in the group with no MetS component, the group with all five MetS components had a higher ferritin level (554.7 (340.1-606.4) vs. 274.2 (198.2-384.4) ng/ml). The odd radio (OR) was higher for MetS in the highest ferritin quartile (OR = 2.29, 95% CI = 1.47-3.54) compared with the lowest ferritin quartile after adjustment for multi-factors. After 4-year follow up, 79 subjects newly diagnosed with MetS in 857 cohort male participants in 2013. Compared with the lowest ferritin quartile, the RR of the highest ferritin quartile was 2.55 (95% CI = 1.30-5.00) after multiple adjustments (*p* < 0.01).

**Conclusion:**

Our findings confirm that the serum ferritin level is associated with the independent components of MetS, and elevated ferritin level is an independent risk factor for MetS development in the Chinese male population during the 4-year follow-up period.

## Background

Metabolic Syndrome (MetS) is a series of metabolic abnormalities, whose clinical features are based on central obesity, insulin resistance, hypertension, high triglycerides, low high-density lipoprotein cholesterol (HDL), and the decline of glucose tolerance or type 2 diabetes mellitus [1]. There is much information indicating that each abnormality involved in the MetS can increase the morbidity and mortality of cardiovascular diseases and cancers [2-4]. From the studies of investigating factors associated with MetS, we have known that multiple factors, such as high uric acid level and inflammatory cytokines, are closely associated with MetS [5,6]. Although the etiology of MetS and the mechanism researches have made great progress, further research is still necessary because of their complexity.

Serum ferritin is a kind of storage of iron in the body tissue soluble protein, which is widely used as a proxy variable to reflect body iron accumulation in healthy individuals [7]. Body iron is a strong pro-oxidant which causes oxidative tissue damage by catalyzing the formation of free radicals of several cellular reactions [8-10]. Oxidative stress was indicated to regulate ferritin mRNA, protein level and its secretion [11,12]. Excessive accumulation of serum ferritin can lead to pathological change in the liver [13], circulatory [14] and musculoskeletal system [15].

Besides, increasing serum ferritin level plays an important role in metabolic risk factors such as type 2 diabetes [16], insulin resistance [17], hypertension [18], and dyslipidemia [19,20]. Increasing evidence showed that elevated serum ferritin is associated with MetS [21]. A study proposed by Abril-Ulloa indicated an observation that there was a higher prevalence of MetS among participants with higher ferritin level [22]. However, the exact molecular mechanism of the relationship between serum ferritin level and MetS remains unclear.

To our knowledge, there was no prospective study on the relationship between the serum ferritin level at baseline and the development of MetS in China so far. Additionally, in our previous studies from Fangchenggang [23], a coastal city in the southwest of China, we found that the average serum ferritin level was more than twice as that of other places in China [24], also America [25] and Korea [26]. It is unknown whether abnormally high ferritin concentrations are associated with MetS or not. Accordingly, we carried out a cross-sectional and longitudinal study to evaluate the temporal relationship between the serum ferritin level at baseline and the risk of MetS in Fangchenggang.

## Methods

### Study populations

A population-based study, which is called the Fangchenggang Area Males Health and Examination Survey (FAMHES) among Chinese males aged from 17–88 years in Guangxi, was designed to investigate the effects of environment, genetic factors and their interaction on the development of age-related chronic diseases [27]. All participants provided written informed consents, and the study was approved by the Ethics and Human Subject Committee of Guangxi Medical University.

The cross-sectional study was carried out among 4303 participants who completed a large-scale comprehensive demographic survey by a face-to-face interview and professional health physical examination by trained physicians at the Medical Centre of Fangchenggang First People’s Hospital between September 2009 and December 2009. Of the 4303 candidates, we excluded participants who met following criteria: [1] missing serum ferritin result, [2] having haemochromatosis caused by the abnormal high level of serum ferritin, [3] knowing a past history of malignancy, liver disease, nephropathy, dyslipidemia or/and thyroid parafunction, [4] alcohol intake 12 h before physical examination. In the end, 2417 unrelated participants (56.17%) aged 20–73 years old were enrolled into the cross-sectional study.

Based on the cross-sectional study, we excluded participants who met following criteria: [1] diagnosed as baseline MetS, [2] missing communication, [3] unwillingness or having some diseases unsuited to participate in the follow-up, 1109 participants (45.88%) were selected into the longitudinal study, and the follow-up duration is 4 years.

### Data collection and definitions

The data of this study were collected through using a standardized questionnaire, which included demographic characteristics (age, ethnicity, occupation, education, etc.), lifestyle characteristics (smoking status, alcohol intake, physical activity, etc.), health status, and medical history, according to a face-to-face interview by trained physicians. Smoking status was classified: when the history of smoking arrived at even more than six months, it was defined as a smoker, otherwise, it was called nonsmoker. Alcohol drinking was defined as ethanol intake once or more each week [28]. Participants who did sports two hours or more than it per week were considered have physical activity [29]. Family history of chronic diseases was defined the participants’ parents or siblings had a history of one of the following diseases: hypertension, type 2 diabetes mellitus, coronary heart disease or stroke [27]. Waist circumference was measured the participant’s natural waist. Blood pressure was measured by using a mercury sphygmomanometer on the right aim of participants who took a 5 minutes rest before measurement according to the world health without shoes by trained nurses. Then, body mass index (BMI) was calculated by the body weight (Kg) divided by the square of height (m^2^).

### Clinical and laboratory measurements

The blood specimens used for biochemical analysis came from the participants who had been told on an empty stomach before drawing blood in the physical examination department of Fangchenggang First People’s Hospital. Afterwards, the frozen blood samples which had been centrifuged 20-25minutes and stored at 80 degrees below zero until analysis were transported to the First Affiliated Hospital of Guangxi Medical University clinical laboratory in Nanning within two hours. And the data of triglyceride, high-density lipoprotein, and serum glucose were collected by using Dimension-Rex Chemistry Analyzer (Dade Behring) to analysis in the Clinical laboratory of Fangchenggang First People’s Hospital. Besides, the serum ferritin was measured by electrochemiluminescence immunoassay on the COBAS 6000 system E601 (Elecsys module) immunoassay analyzer (Roche Diagnostics, GmbH, Mannheim, Germany) with the same batch of reagents.

### Definition of MetS cases

The MetS was defined based upon the updated National Cholesterol Education Program Adult Treatment Panel III for Asian Americans [30] as having arbitrarily three or more components in following:[1] waist circumference reach 90 cm or more, [2] triglyceride at least 1.7 mmol/L, [3] HDL under 1.03 mmol/L, [4] blood pressure at least 130 mmHg in systolic blood pressure/85 mmHg in diastolic blood pressure or current use of hypotensive drugs or having the history of hypertension, [5] fasting glucose attain 5.6 mmol/l or previously diagnosed with type 2 diabetes mellitus or taking medications to control hyperglycaemia.

### Statistical analysis

**Figure 1:**
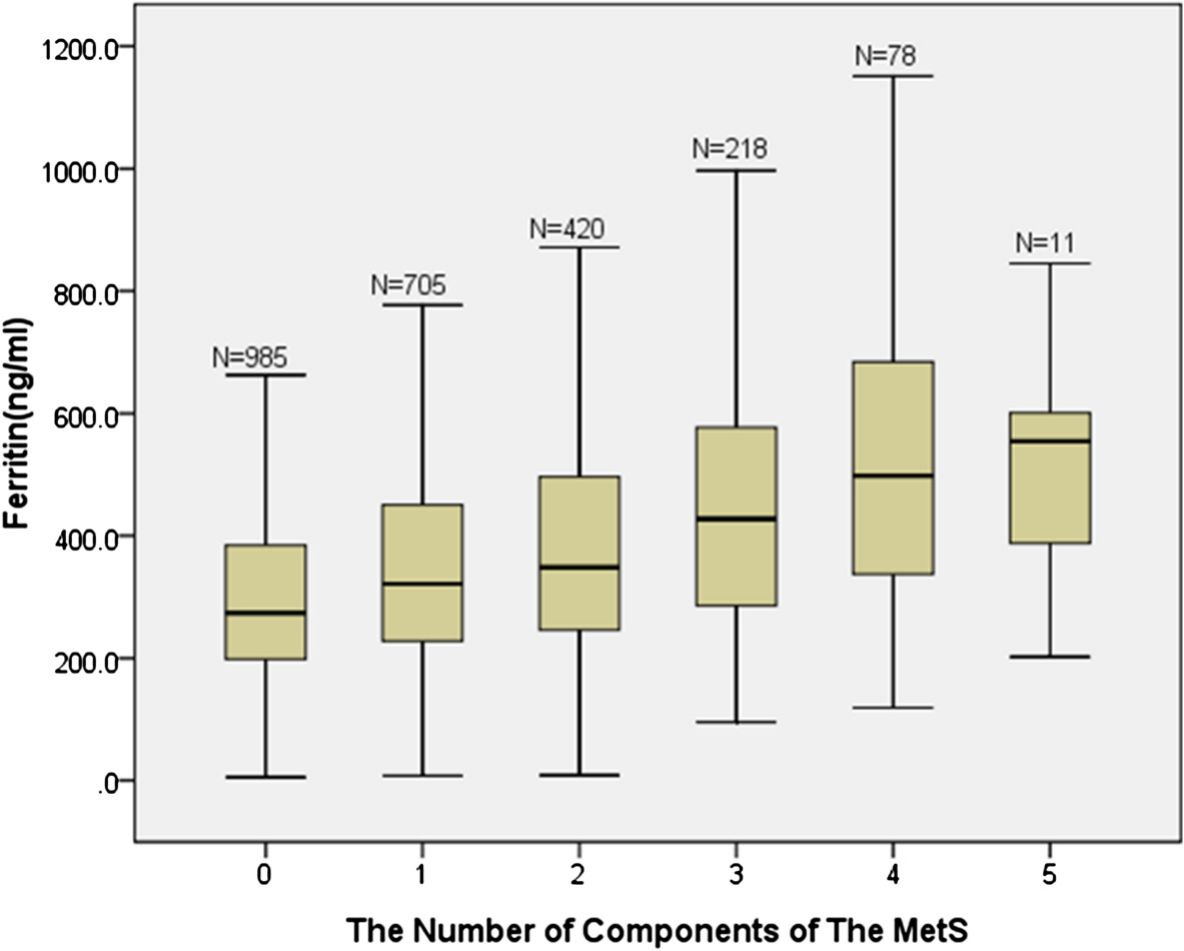
**Distribution of ferritin levels according to the numbers of components of the MetS.** The ferritin level had a growing trend when the number of MetS components increased in groups (*p* < 0.001).The bars represent median, 25th, and 75th percentile of ferritin.

The data of the study came from 2417 individuals aged 18–63 years medians (interquartile range) for continuous variables, counts (percentages) for categorical variables in the baseline data. Mann–Whitney U test of nonparametric test was used to continuous variables and x^2^ test to compare categorical variables. Spearman correlation was used to analyze the correlation coefficient between serum ferritin and individual components of the metabolic syndrome. Unadjusted as well as adjusted odds radio (ORs) and 95% confidence interval (95% CI) for metabolic syndrome and its components in the quartile group of serum ferritin were calculated by Binary logistic regression models. The ORs calculations were age, smoking status, alcohol stake, family history of chronic diseases, and physical activity. The x^2^ statistic was used to obtain the probability value for the trend in Figure 1. Binary logistic regression model was also used to compute relative risks (RRs) and 95%Cl for MetS according to quartiles of serum ferritin. Statistical analyses were performed by SPSS 16.0 for windows, all tests were two-sided, and statistical significance was set to *p* < 0.05.

## Results

### Selection of study participants

**Figure 2:**
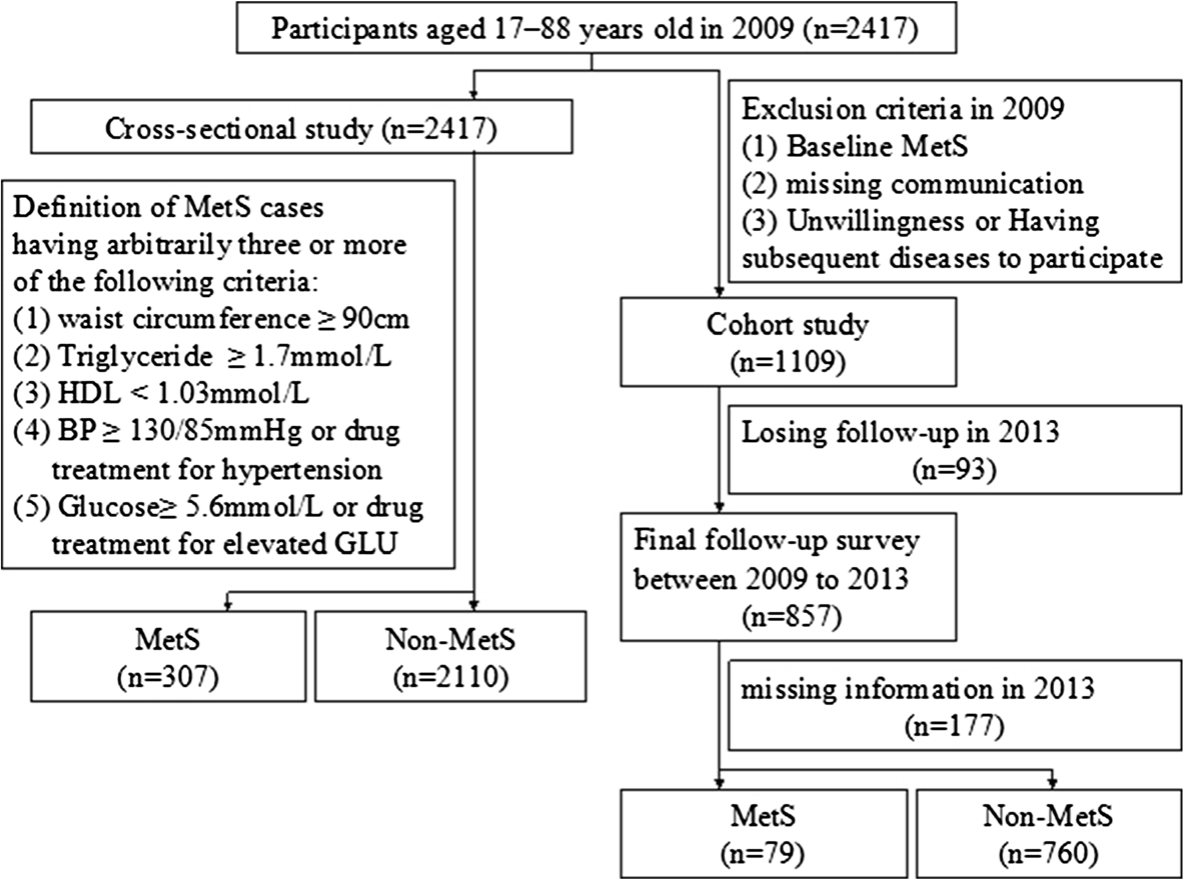
**Flow chart for selection of study participants.** Based on the inclusion criteria, 2417 male participated in the cross-sectional study and 1009 male participated in the cohort study.

Figure 2 shows the flowchart for participants’ selection of the study. 2417 eligible male subjects were recruited, including 307 participants with MetS and 2110 participants without MetS (nonMetS). Among the 2417 subjects, 1109 subjects were selected in cohort study. 93 participants lost follow-up in 2013 were excluded in the cohort study. Following on a quadrennial basis for 4 years using a protocol similar to the initial examination, 177 subjects missed record in anthropometric measurements and clinical biochemistry assays. Eventually, 839 participants were enrolled for final cohort study analysis and observed for the development of MetS.

### General characteristics in cross-sectional survey

Table 1 shows the demographic, clinical and laboratory characteristics of 2417 participants. Data are presented for 307 subjects with MetS and 2110 without MetS (nonMetS), and the incidence of MetS in 2009 is 12.70%. The ferritin level of MetS group was obviously higher than that of the nonMetS group (median and percentiles 25–75: 447.4 (294.1-612.4) vs. 302.4 (215.0-435.8) ng/ml, *p* < 0.01). Besides, compared with nonMetS group, MetS group had higher median age, blood arterial pressure, waist circumference, triglyceride, glucose, BMI, and lower HDL (all *p* < 0.01). However, no association was found between MetS and smoking consumption (*p* = 0. 08), physical activity (*p* = 0. 10).

**Table 1:** **General characteristics of study population stratified for MetS and non-MetS in the cross-sectional study**

	**Total**	**MetS**	**Non-MetS**	**p**
No. of subjects	2417	307	2110	
Age (years)	36(29–44)	43(35–48)	35(28–43)	<0.01
Ferritin (ng/mL)	316.8(221.4-459.9)	447.4(294.1-612.4)	302.4(215.0-435.8)	<0.01
SBP (mm Hg)	120(110–126)	130(120–140)	116(108–122)	<0.01
DBP (mm Hg)	78(70–80)	86(80–90)	76(70–80)	<0.01
Waist circumference (cm)	80.5(73.4-87.5)	92.4(88.0-95.9)	79.0(72.5-85.5)	<0.01
Triglycerides (mmol/L)	1.13(0.78-1.76)	2.50(1.88-3.69)	1.03(0.74-1.49)	<0.01
Glucose (mmol/L)	5.2(4.8-5.6)	5.8(5.4-6.2)	5.1(4.8-5.5)	<0.01
HDL (mmol/L)	1.35(1.18-1.57)	1.16(1.01-1.38)	1.38(1.21-1.59)	<0.01
BMI (kg/m2)	23.08(20.80-25.52)	27.05(25.06-28.74)	22.62(20.55-24.78)	<0.01
Smoking status, yes, n (%)	1248(51.6%)	173(56.4%)	1075(50.9%)	0.08
Alcohol drinking, yes, n (%)	972(40.2%)	144(46.9%)	828(39.2%)	0.01
Physical activity, yes, n (%)	618(25.6%)	90(29.3%)	528(25.0%)	0.10
Family history of chronic disease, yes, n (%)	485(20.1%)	85(27.7%)	400(19.0%)	<0.01

Data are presented as median (25 percenile, 75 percentile) or counts (percent).

The Chi-square test for categorical variables and The Mann-Whitrey U test for continuous variables.

SBP, systolic blood pressure; DBP, diastolic blood pressure; HDL, high density lipoprotein cholesterol; BMI, body mass index; MetS, metabolic syndrome.

### Correlation coefficients between ferritin and metabolic risk parameters

Spearman correlation coefficients were revealed ferritin and individual components of MetS, age and BMI in Table 2. There is a significant positive correlation between ferritin and SBP, DBP, waist circumference, triglyceride, glucose, low HDL, age, and BMI (all *p* < 0.001). The strongest correlation (R = 0. 333) was shown between ferritin and waist circumference.

**Table 2:** **Spearman correlation coefficients between ferritin and metabolic risk parameters**

**Variables**	**R**	***p***
SBP	0.110	<0.001
DBP	0.158	<0.001
Waist circumference	0.333	<0.001
Triglyceride	0.315	<0.001
Glucose	0.089	<0.001
HDL	−0.130	<0.001
Age	0.109	<0.001
BMI	0.302	<0.001

A positive correlation between the number of MetS components and a higher level of ferritin was indicated in Figure 1. The groups with 0, 1, 2, 3, 4, 5 of MetS components had 985, 705, 420, 218, 78, 11 subjects and 274.2, 320.9, 347.9, 427.5, 497.7, 554.7 ng/ml of median ferritin level respectively.

### Association between serum ferritin concentrations and MetS and its individual components

As shown in Table 3, increasing ORs for MetS were observed from 1^st^ to 4^th^ ferritin quartiles by binary logistic regression. Compared with the lowest quartile of ferritin (1^st^ Q), the ORs and 95% CI of MetS with respect to 2^nd^ Q, 3^rd^ Q, 4^th ^Q were 1.48 (0.95-2.29), 2.08 (1.37-3.16), 5.12 (3.48-7.52) respectively in the unadjusted analyses. After adjusting for age, the ORs and 95% CI of MetS with respect to 2^nd^ Q, 3^rd^ Q, 4^th^ Q were 1.61 (1.03-2.52), 2.21 (1.44-3.37), 5.08 (3.44-7.50) respectively in model 1. The ORs and 95% CI of MetS with respect to 2nd Q, 3rd Q, 4th Q were 1.59 (1.01-2.48), 2.11 (1.37-3.22), 4.90 (3.31-7.26) respectively in model 2, plus model 1 and adjusted physical activity, family history of chronic diseases (4 covariate: hypertension, diabetes mellitus, stroke, coronary heart disease), lifestyle factor of alcohol drinking status and smoking status. The ORs and 95% CI of MetS with respect to 2^nd^ Q, 3^rd^ Q, 4^th^ Q were 1.20 (0.77-2.08), 1.32 (0.83-2.12), 2.29 (1.47-3.54) respectively in model 3, plus model 2 and further adjusted BMI.

**Table 3:** **Odd radios and 95% confidence interval of metabolic syndrome according to quartiles of ferritin: a binary logistic regression**

					**OR (95% CI)**				
**Ferritin**	**Cases (%)**	**Unadjusted**	***p1***	**Model 1**	***p2***	**Model 2**	***p3***	**Model 3**	***p4***
1st Q	36(5.97%)	Ref		Ref		Ref		Ref	
2nd Q	52(8.60%)	1.48(0.92-2.29)	0.08	1.61(1.03-2.52)	0.04	1.59(1.01-2.48)	0.04	1.27(0.77-2.08)	0.35
3rd Q	71(11.72%)	2.08(1.37-3.16)	<0.01	2.21(1.44-3.37)	<0.01	2.11(1.37-3.22)	<0.01	1.32(0.83-2.12)	0.25
4th Q	148(24.54%)	5.12(3.48-7.52)	<0.01	5.08(3.44-7.50)	<0.01	4.90(3.31-7.26)	<0.01	2.29(1.47-3.54)	<0.01
*P*_*trend*_		<0.01		<0.01		<0.01		<0.01	

1st Q: <221.4 ng/ml; 2nd Q: 221.4-318.7 ng/ml; 3rd Q: 318.8-459.9 ng/ml; 4th Q: >459.9 ng/ml.

Model 1,Odds radio adjusted for age, model 2 plus model 1 and physical activity, family history of chronic diseases(4 covariates: hypertension, diabetes mellitus, stoke, coronary heart disease), lifestyle factor of alcohol drinking status and smoking status. model 3 further BMI.

**Figure 3:**
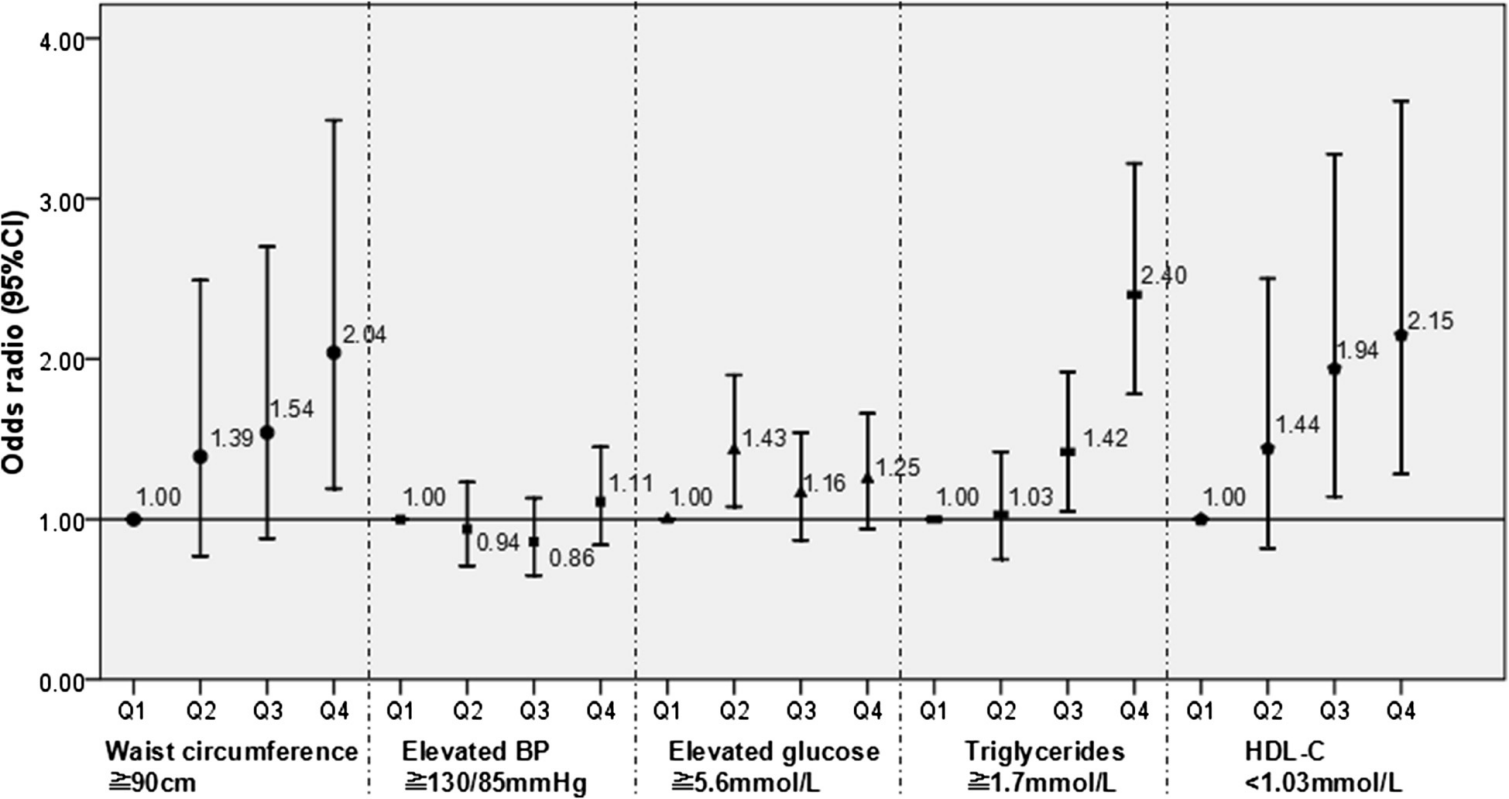
**Multiple adjusted odds radio and 95% CI of individual components of MetS according to quartile of ferritin.** The OR values increased with increasing quartile group of serum ferritin concentrations in waist circumference, triglycerides, and low HDL (all *p* < 0.01). The serum ferritin level was associated with MetS components adjusting age, BMI, smoking status, alcohol stake, family history of chronic diseases.

What’s more, Figure 3 provides the association of ferritin and individual components of MetS. These analyses adjusted age, BMI, physical activity, family history of chronic diseases (4 covariate: hypertension, diabetes mellitus, stroke, coronary heart disease), lifestyle factor of alcohol drinking status and smoking status. Compared with the lowest ferritin quartile, the ORs and 95% CI of waist circumference, triglyceride, HDL in the highest ferritin quartile were significant 2.04 (1.19-3.49), 2.40 (1.78-3.22), 2.15 (1.28-3.61), respectively.

### Baseline characteristics in cohort study

857 participants followed up for 4 years were selected from 2110 nonMetS in 2009 (Table 4). 18 missed lifestyle questionnaire or lacked the data of diagnosing MetS. Data are presented for 79 subjects with MetS and 760 without MetS, and the new incidence of MetS in 2013 is 9.42%. The ferritin level of MetS group was obviously higher than that of the nonMetS group (357.1 (260.0-526.1) vs. 293.4 (208.4-417.0) ng/ml, *p* < 0.01). Besides, MetS subjects had higher age, blood arterial pressure, waist circumference, triglyceride, glucose, BMI, and lower HDL than no MetS subjects (all *p* < 0.01). However, no association was found between MetS and smoking consumption, physical activity.

**Table 4:** **Baseline characteristics of study population stratified for the MetS and non-MetS in 2013**

	**Total**	**MetS**	**Non-MetS**	***p***
No. of subjects	839	79	760	
Age (years)	37(30–45)	40(35–45)	37(29–45)	0.01
Ferritin (ng/mL)	301.1(210.9-426.6)	357.1 (260.0-526.1)	293.4(208.4-417.0)	<0.01
SBP (mm Hg)	117(108–124)	120(110–126)	116(108–124)	<0.01
DBP (mm Hg)	78(70–80)	80(74–84)	76(70–80)	<0.01
Waist circumference (cm)	79.1(72.5-85.3)	87.0(82.8-90.4)	78.0(72.0-84.2)	<0.01
Triglyceride (mmol/L)	1.01(0.72-1.46)	1.52(1.29-2.06)	0.95(0.69-1.38)	<0.01
Glucose (mmol/L)	5.1(4.8-5.5)	5.3(5.0-5.6)	5.1(4.8-5.4)	<0.01
HDL (mmol/L)	1.40(1.22-1.61)	1.21(1.11-1.32)	1.41(1.24-1.63)	<0.01
BMI (kg/m2)	22.71(20.61-24.71)	25.46(23.74-27.27)	22.40(20.43-24.25)	<0.01
Smoking status, yes, n (%)				0.35
Smoking	404(48.2%)	42(53.2%)	362(47.6%)	
Non-smoking	435(51.8%)	37(46.8%)	398(52.4%)	
Alcohol drinking, yes, n (%)	337(40.2%)	31(39.2%)	306(40.3%)	0.86
Physical activity, yes, n (%)	185(22.1%)	23(29.1%)	162(21.3%)	0.11
Family history of chronic disease, yes, n (%)	156(18.6%)	16(20.3%)	140(18.4%)	0.69

Data are presented as median (25 percenile, 75 percentile) or counts (percent).

The Chi-square test for categorical variables and The Mann-Whitrey U test for continuous variables.

SBP, systolic blood pressure; DBP, diastolic blood pressure; HDL, high density lipoprotein cholesterol; BMI, body mass index; MetS, metabolic syndrome.

### Association between serum ferritin concentrations and the incidence of the MetS after a 4-year follow up

In the final analyses (Table 5), compared with the lowest quartile of ferritin, in the unadjusted analyses, the RR of MetS in the highest ferritin quartile was 2.50 (1.28-4.88) (*p* = 0. 07). Furthermore, model 1 adjusted age, the RR of MetS in the highest ferritin quartile was 2.58 (1.32-5.05) (*p* < 0.01). Eventually, model 2 adjusted physical activity, family history of chronic diseases (4 covariate: hypertension, diabetes mellitus, stroke, coronary heart disease), lifestyle factor of alcohol drinking status and smoking status, the RR of MetS in the highest ferritin quartile was 2.55 (1.30-5.00) (*p* < 0.01).

**Table 5:** **RRs and 95% CI for the incidence of the MetS according to quartiles of ferritin level after 4-year follow-up**

				**RR (95% CI)**			
**Ferritin**	**Cases (%)**	**Unadjusted**	***p1***	**Model 1**	***p2***	**Model 2**	***p3***
1st Q	12(5.74%)	ref		ref		ref	
2nd Q	15(7.14%)	1.26(0.58-2.77)	0.56	1.36(0.62-2.99)	0.45	1.33(0.60-2.95)	0.48
3rd Q	22(10.43%)	1.91(0.92-3.97)	0.08	2.03(0.97-4.24)	0.06	2.01(0.96-4.22)	0.07
4th Q	30(14.35%)	2.75(1.37-5.54)	<0.01	2.87(1.42-5.79)	<0.01	2.84(1.40-5.75)	<0.01
*P*_*trend*_		<0.01		<0.01		<0.01	

1st Q: <210.9 ng/ml; 2nd Q: 210.9-301.0 ng/ml; 3rd Q: 301.1-426.6 ng/ml; 4th Q: >426.6 ng/ml.

model 1 were adjusted age, model 2 further adjusted smoking status, alcohol drinking status, family history of chronic disease, physical activity.

## Discussion

In our study, the data was observed an evident correlation between serum ferritin concentrations and the independent components of MetS. Blood pressure, waist circumference, fasting glucose, triglyceride, low HDL, age, and BMI were positively correlated with ferritin concentrations. What’s more, waist circumference and triglycerides were the best correlates of serum ferritin, and this suggested high ferritin concentrations may reflect hypertriglyceridemic phenotype which was predicted in women by low lipoprotein(a) levels [31].

Besides, the median of ferritin level had a growing trend when the number of MetS components increased in groups. Similarly, a previous study by Jiang Li et al. had reported that with the higher ferritin level quartile, the higher percentage of participants with MetS or its components [32]. In addition, several studies have reported the association between serum ferritin concentrations and MetS diagnostic components. In accordance with a 5-year prospective cohort in Korean men [33], we also found it had no appreciable effect on the relationship between serum ferritin level and waist circumference after adjusted for BMI. A report by Mateo-Gallego et al. has shown the serum ferritin level is a major determinant of hypertriglyceridemia in individuals with familial-type dyslipidemia [34]. Our results support the supposition that serum ferritin level is correlated with increasing triglyceride concentrations in Chinese males. The same result also shows on the association between ferritin level and HDL.

Sung Keun Park et al. found a longitudinal relationship between serum ferritin and the development of MetS in 13084 Korean males from a 5–year follow-up study [26]. Our results are in line with cross-sectional study and also extend these observations to examine the prospective association between baseline ferritin level and the development of MetS over a 4-year follow-up study. In our study, we found that the highest quartile of ferritin level had significant relationship with the development of MetS, and the risk of MetS increased with increasing quartile group of serum ferritin concentrations. However, we could not reach the level of a significant result after adjustment for BMI, and It maybe there were strong associations between BMI and the complements of MetS, such as waist circumference.

Interestingly, although the ferritin concentrations of participants are higher than that in previous studies, the incidence of MetS in the study in 2009 was 12.70%, which was similar to that in Chinese mainland in 2004 (13.7%). Jung-Su Chang reported the incidence of MetS was 43.1% in a 1260 Taiwanese men study where the mean of ferritin level is 229 (±349) ng/ml [35]. Taiwan is a sea island, and the age distribution of male and female participant trend towards middle-aged (mean and standard deviation: 54.3 ± 17.8 yrs old) in the study. However, the age distribution of our participants is skewed distribution which mainly concentrated in the young stage (median and percentiles 25–75: 36 (29–44) yrs old). That may indicate the MetS incidence increases with age.

Environmental factors and genotype could make an approximately equal contribution to the body iron status [36], and serum ferritin level differs visibly according to age, gender, geographic location, and ethnicity [37,38]. Harris and colleagues found Asian men and women have higher adjusted mean serum ferritin concentrations compared with their white counterparts [39]. Fangchenggang is a littoral city in which diet and lifestyle are different from the inland and island. Long-time constant diet or lifestyle in causing the increase of serum ferritin level may lead to some changes of serum ferritin level and its mechanism in the body as well. Moreover, after 4-year follow-up period, we found that the highest level of ferritin was associated in 2.55-fold to the development of MetS. There were 79 participants new suffering MetS, from 857 which were selected out from 2417 nonMetS in 2009, and we couldn’t get a comprehensive result because of selection bias. The follow-up participants had a younger age distribution, and they were randomly selected from nonMetS participants in 2009, so the risk of suffering MetS was smaller. However, the incidence of MetS was 9.42%, which indicated MetS is rapidly developing in Fangchegngang. It’s prominent that we should pay more attention on the prevention of MetS.

### Limitations

Nevertheless, when interpreting the findings of the current work, some limitations should be considered. Firstly, the female subjects were not included in the participants in our study, and our results may lack of enough persuasion and not be extrapolated to females. Secondly, we couldn’t analyze comprehensively the association of serum ferritin and MetS, due to lack of other iron-related markers such as trans-ferritin and total iron-binding capacity as a marker of body iron status. Thirdly, the information for dietary intake is not concluded, which may influence body iron stores.

## Conclusions

Our findings have explicitly shown that the elevated ferritin level is an important independent risk factor for the development of MetS in the littoral Chinese male population. Serum ferritin level may become a new guidance of the early prevention of MetS. However, the molecule mechanism of ferritin playing in the development process of MetS is still unclear and it needs further research to explore.

## Abbreviations

MetS: Metabolic syndrome
FAMHES: Fangchenggang area males health and examination survey
HDL: High-density lipoprotein cholesterol
SBP: Systolic blood pressure
DBP: Diastolic blood pressure
BMI: Body mass index
OR: Odd radio
95%CI: 95% confidence interval
RR: The relative risk
